# Transport property of multi-band topological material PtBi$_2$ studied by maximum entropy mobility spectrum analysis (MEMSA)

**DOI:** 10.1038/s41598-021-85364-6

**Published:** 2021-03-18

**Authors:** Haijun Zhao, Wenchong Li, Yue Chen, Chunqiang Xu, Bin Li, Weidong Luo, Dong Qian, Zhixiang Shi

**Affiliations:** 1 School of Physics, Southeast University, Nanjing, 211189 China; 2 Information Physics Research Center, Nanjing University of Posts and Telecommunications, Nanjing, 210023 China; 3 Key Laboratory of Artificial Structures and Quantum Control (Ministry of Education), School of Physics and Astronomy, Shanghai Jiao Tong University, Shanghai, 200240 China; 4 Tsung-Dao Lee Institute, Shanghai Jiao Tong University, Shanghai, 200240 China

**Keywords:** Superconducting properties and materials, Topological matter

## Abstract

Electrical transport of both longitudinal and transverse directions carries rich information. Mobility spectrum analysis (MSA) is capable of extracting charge information from conductivity tensor, including charge types, concentration and mobilities. Using a numerical method based on maximum entropy principle, i.e., maximum entropy mobility spectrum analysis (MEMSA), mobility spectrum for $\beta$-type PtBi$_2$ is studied. Three hole-pockets and two electron-pockets were found, including a small hole pocket with very high mobility, which is very likely corresponding to Dirac Fermions. Benefiting from our high resolution result, we studied temperature dependence of carrier properties and explained the sign change phenomenon of Hall conductivity. We further compared the results with band structure obtained by our first principle calculation. The present results prove MEMSA is a useful tool of extracting carries’ information in recently discovered Iron-based superconductors, and topological materials.

## Introduction

Extracting information of charge carriers is an important topic in condensed matter physics. Despite its well development within semiconductor field, recent studying of Iron-based superconductors (IBSC), and topological materials generates new challenges. Unlike copper oxides, the transport properties of IBSC show rich physics due to their complex d-orbital energy bands with unique topology^1^. Beside the unconventional high temperature superconductivity^2^, other properties, such as the quantum transport phenomena resulting from Dirac-cones forming^3,4^, have also attracted many focuses. Various experimental observations and theoretical calculations reveal the multi-band nature of IBSCs. However, debates continue on understanding the real band picture of IBSCs, and it is very challenging to experimentally differentiate the various Fermi pockets in these complex multiple-band materials, where both electrons and holes with different effective masses are present in momentum space. Moreover, the existence Dirac-like quantum states, which contain carriers with extremely small concentration, but markedly high mobilities, makes the problem even more interesting.

Topological materials have also attracted considerable attention not only in the condensed matter physics but also in the applied science society due to their novel properties and potential applications^5–9^. Two examples of topological semimetals are: Dirac semimetals, which possess fourfold degenerate band crossings in momentum space, and Weyl semimetals, in which the spin degeneracy is lifted. In the latter case, the band crossings are referred to as Weyl points^10–12^. Recently, several new types of Topological semimetals identified by threefold, sixfold, eightfold band crossings near the Fermi level were proposed^13^. In particular, the threefold point fermions have been indicated might exist in the materials with WC-type structure, such as MoP, WC, TaN and ZrTe^10,14,15^, which can be viewed as an intermediate state between fourfold degenerate Dirac points and twofold degenerate Weyl points.

Experimentally, the carrier property can be measured by angle-resolved photoemission spectroscopy (ARPES). Benefitting from this measurement, the first experimental observations of the Dirac-cone states in IBSC were successfully made^16^. For topological materials, the threefold fermions have been demonstrated in MoP^17^. However, the energy resolution of ARPES is around mev, which limits its ability of separating different bands. Quantum oscillations are capable of measuring the electronic states at the Fermi level, but are insufficiently sensitive to detect tiny but important pockets, such as Dirac-cone quantum states. In the meanwhile, a relatively large field is required, which may greatly change carriers’ properties, especially for field-sensitive materials, such as Weyl semimetals. Moreover, none of the above measurements can estimate the conductivity contributions from different bands.

Measuring electrical transport of both longitudinal and transverse directions was an important way of obtaining carriers’ type, mobility, and concentration in single band semiconductors. For materials containing one electron band and one hole band, two carrier model was wildly used. However, for multiple-band materials, clear deviation appears. Therefore, new methods are required to fit the measured experimental curves. A straightforward way is hypothesizing both carrier types and number of bands, then, calculating mobility and concentration via fitting^18–20^. In order to avoid the hypothesis, the technique of mobility spectrum (MS), which was initially developed to study carriers in semiconductors^21–24^, was applied to Ba(FeAs)$_2$^25^ and FeSe^26^. The MS resulting from longitudinal and transverse transport under a wide range of magnetic field B up to 50 T, shows a physically reasonable and intrinsic interpretation on the electronic states in low temperature phase. More recently, it was also used to study Type-II Weyl semimetal T$_d$-MoTe$_2$^27^.

Note that, application of MS requires material to have relatively large magnetoresistance. PtBi$_2$ with a layered hexagonal crystal structure was reported to exhibit a large magnetoresistance^28–32^. Both band structures and $Z_2$ invariant calculations suggest PtBi$_2$ as a possible candidate for bulk topological metal^28^. ARPES measurement found a Dirac-cone-like surface state on the boundary of the Brillouin zone, which is identified as an accidental Dirac band without topological protection^29^. Moreover, triply degenerate point (TDP) fermions were predicated by ab-initio calculations, and verified by quantum oscillation^30^. More recently, APRES measurement and first principle calculation detected five bands that could contribute to the TDP^33^. Magnetoresistance measurement for field up to 22T found that magnetoresistance is related to the angle between the magnetic field and the crystalline c axis^32^. In this paper, we use maximum entropy mobility spectrum analysis^34^ (MEMSA) to study carrier properties of PtBi$_2$. The numerical MEMSA method is capable of nicely fit the experimental data (see Figs. S1–S2 of Supplementary Materials). For comparison, We also calculated band structure using first principle calculation. Our results suggest that, MEMSA is a useful tool of detecting carrier type, mobility, and charge concentration in recently discovered Iron-based superconductors, and topological materials.

**Figure 1:**
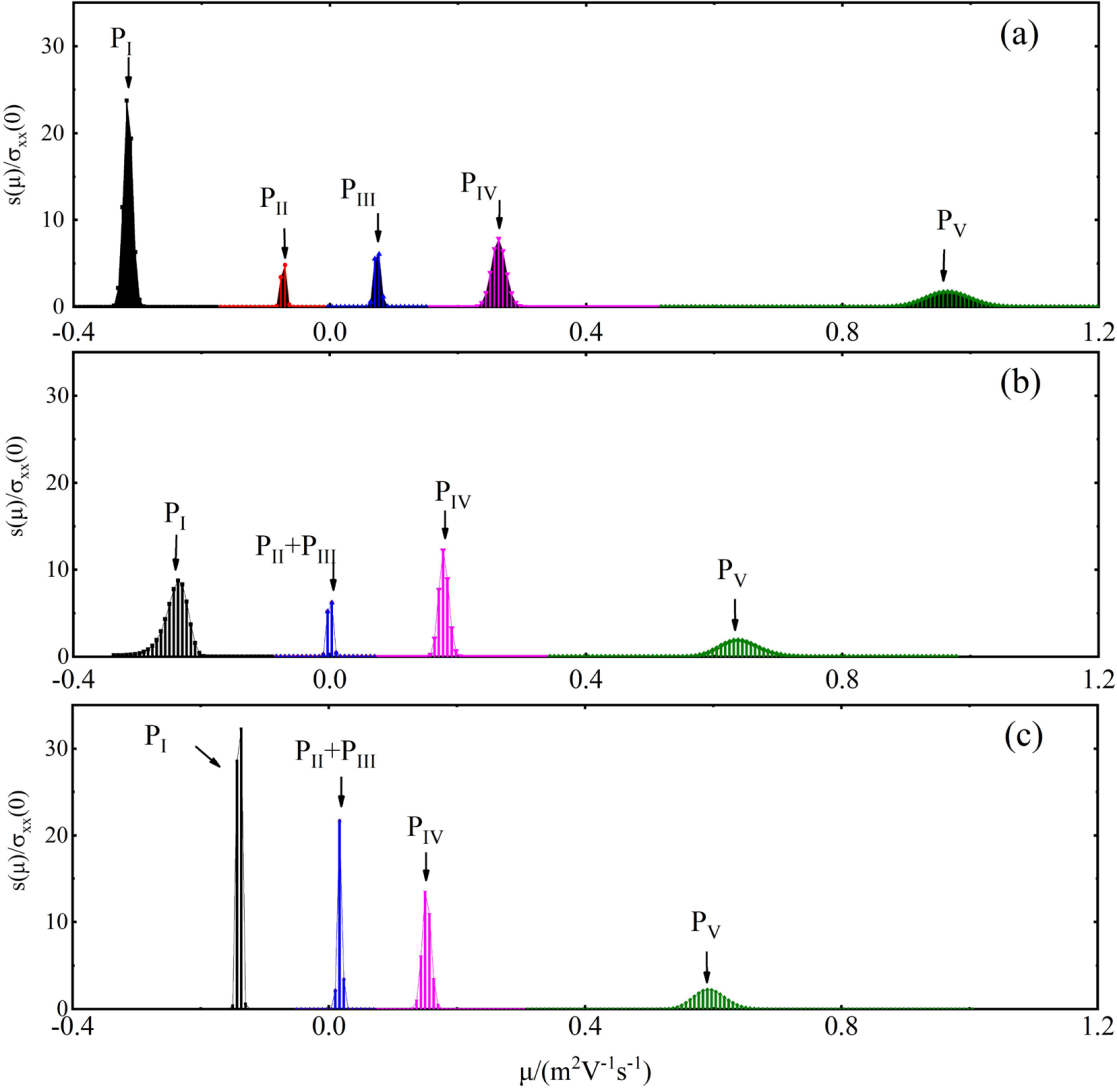
The MS of carriers in PtBi$_2$ for temperature $T=2K$ (**a**), 10*K* (**b**), and 20*K* (**c**), respectively.

**Figure 2:**
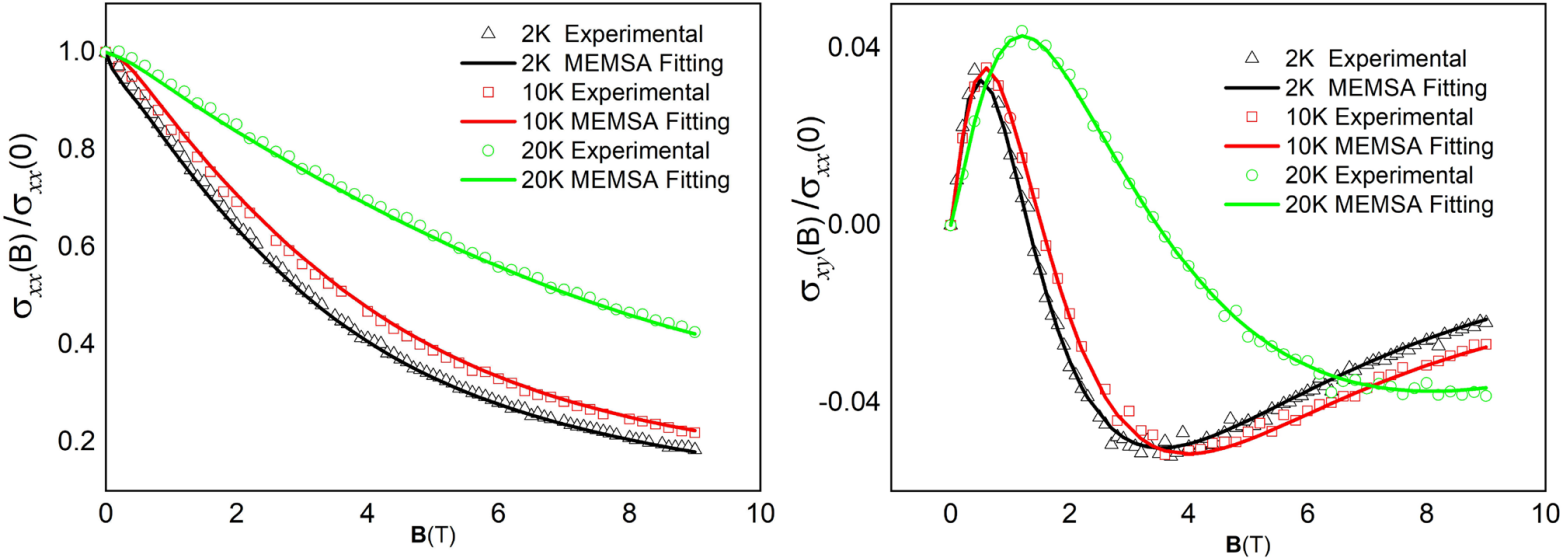
The experimentally measured conductivity (left panel, dots), Hall conductivity (right panel, dots), and their MEMSA fitting (solid lines).

## Numerical algorithm

MEMSA starts from the experimentally measured Magnetoresistivity $\rho _{xx}(B)$ and Hall resistivity $\rho _{xy}= BR_H(B)$, from which, one can calculate the conductivity tensor by:

1 $$\begin{aligned} \sigma _{xx}=\rho _{xx}/(\rho _{xx}^2+\rho _{xy}^2),~ \sigma _{xy}=\rho _{xy}/(\rho _{xx}^2+\rho _{xy}^2). \end{aligned}$$

Within the MS model, the relation between conductivity tensor and mobility is given by^23–26,30,34^:

2 $$\begin{aligned} \sigma _{xx}(B)\!=\!\int ^{+\infty }_{-\infty }\!\!\!\!\dfrac{s(\mu )d\mu }{1+(\mu B)^{2}}, \sigma _{xy}(B)\!=\!\int ^{+\infty }_{-\infty }\!\!\dfrac{\mu Bs(\mu )d\mu }{1+(\mu B)^{2}}, \end{aligned}$$

where the MS is evaluated as follows: $s(\mu )\rightarrow \sum _{j}n_{j}e\mu _{j}\delta (\mu -\mu _{j})$, $n_{j}$ is the concentration of the carriers with mobility $\mu _{j}$. It is assumed that in Eqs. (2), mobilities are negative for electrons and positive for holes.

Mathmatically, Eqs. (2) belong to Fredholm equations of the first kind. MS can be achieved by an inversing method. To reach a high resolution, we use numerical iterations based on maximum entropy principle^24,34^. First, we define the reduced conductivity tensor:

3 $$\begin{aligned}&\overline{\sigma }_{xx}(B_{j})=\frac{\sigma _{xx}(B_{j})}{\sigma _{xx}(0)}\!=\!\sum _{i=1}^{N}\dfrac{p_{i}}{1+(\mu _{i}B_{j})^{2}}\!=\!\sum _{i=1}^{N}K_{ij}^{xx}p_{i}, \end{aligned}$$

4 $$\begin{aligned}&\overline{\sigma }_{xy}(B_{j})=\frac{\sigma _{xy}(B_{j})}{\sigma _{xx}(0)}\!=\!\sum _{i=1}^{N}\dfrac{p_{i}\mu _{i}B_{j}}{1+(\mu _{i}B_{j})^{2}}\!=\!\sum _{i=1}^{N}K_{ij}^{xy}p_{i}. \end{aligned}$$

Calculation of the probability *p* is performed using the Lagrangian multiplier $\lambda$:

5 $$\begin{aligned} p_{i}= & {} \dfrac{exp\{-\sum _{j=1}^{M}(\lambda _{j}^{xx}K_{kj}^{xx}-\lambda _{j}^{xy}K_{kj}^{xy})\}}{Z}, \end{aligned}$$

where $Z=\sum _{k=1}^{N}exp\{-\sum _{j=1}^{M}(\lambda _{j}^{xx}K_{kj}^{xx}-\lambda _{j}^{xy}K_{kj}^{xy})\}$ is the partition function. Assuming $\lambda ^{k+1}=\lambda ^{k}+\delta \lambda ^k$, and $\delta \lambda ^k<<\lambda ^{k}$, it can be proved that $\sigma ^{k+1}=\sigma ^{k}-A^{k}\delta \lambda ^{k}$, where, $\sigma =[\overline{\sigma }_{xx},\overline{\sigma }_{xy}]$, the matrix $A^{k}$ is given by:

6 $$\begin{aligned} A^{k}_{ju}= & {} \sum _{k=1}^{N}K_{ij}P^{k}_{i}K_{iu}. \end{aligned}$$

Replacing $\sigma ^{k+1}$ with experimental data, one have:

7 $$\begin{aligned} \delta \lambda ^{k}= & {} -(A^{k})^{-1}\sigma ^{k}-\overline{\sigma }_{exp}. \end{aligned}$$

The matrices $A^{k}$ were inverted using singular value decomposition. Eq. (7) gives the approximate difference between the new value of $\lambda ^{k+1}$ and the old one $\lambda ^{k}$. The Lagrangian multipliers can be found using the following numerical iterative procedure: First, assume a group of initial values of $\lambda$, and use Eq. (5) to calculate the probability *p*; second, calculate the corresponding conductivity matrix $\sigma$ (Eqs. (3-4) ) and matrix *A* (Eq. (6)); third, use Eq. (7) to calculate the modification of Lagrangian multipliers and calculate new set of Lagrangian multipliers $\lambda ^{k+1}=\lambda ^{k}+\alpha \delta \lambda ^k$, where $0<\alpha \le 1$. The loop continues until each element of $\delta \lambda ^k$ is sufficiently small. The corresponding MS can be calculated from Eq. (5).

## Results and discussion

In Fig. 1a–c, we show MS for temperature $T=2$ K, 10 K, and 20 K, respectively. The corresponding fitting to the conductivity tenser, as well as measured experimental data are shown in Fig. 2. It is clear that comparing previous methods^25,27^, our MEMSA method perfectly fits the experimental data of conductivity $\sigma _{xx}$ and Hall conductivity $\sigma _{xy}$ spontaneously. The resolution is greatly improved, resulting in well separation of peaks on the MS curve. Each of these peaks are corresponding to an electron or a hole pockets. For a specific pocket *i*, we calculate the following three values: I. the ratio of conductivity contribution at zero field $\gamma _i$; II. the location of the peak, i.e., average mobility $\mu _i$; and III. carrier concentration *n*. Note that, small peaks whose contribution $\gamma$ ($<4\%$) are neglected, since they might be generated by white noises in the experiment. For $T=2$ K, two electron and three hole pockets are identified (see Fig. 1a). The number of bands is agree nicely with ARPES measurements^33^ and first principle calculations^30,33^. The carriers’ concentration are $n_I=4.5\times 10^{26}\,\mathrm{m}^{-3}$ for electron band $P_I$, $n_{II}=2.8\times 10^{26}\,\mathrm{m}^{-3}$ for electron band $P_{II}$, $n_{III}=3.9\times 10^{26}\,\mathrm{m}^{-3}$ for hole band $P_{III}$, and $n_{IV}=2.8\times 10^{26}\,\mathrm{m}^{-3}$ for hole band $P_{IV}$. Electron band $P_{V}$ is of one order smaller ($n_{V}=2.8\times 10^{26}\,\mathrm{m}^{-3}$), but its mobility is high, leading to a relatively large conductivity contribution ($15\%$). In turn, it is very likely corresponding to a Dirac-cone pocket that was previously reported^31^. The summation of concentration for electrons $n_e=7.3\times 10^{26}\,\mathrm{m}^{-3}$, whereas, for holes, $n_h=7.2\times 10^{26}\,\mathrm{m}^{-3}$. Those values are similar to Pyrite PtBi$_2$^35^, and many other reported large magnetoresistance topological materials (e.g. WTe$_2$^5^ and LaSb^6^), but is higher than Cr$_3$As$_2$^8^. Note that, $n_h\approx n_e$, which is similar to Pyrite PtBi$_2$^35^ and WTe$_2$^5^, indicating that, the conductivity contribution of electrons and holes are comparable when their mobility difference is small. For $T=10K$, all the peaks move toward low mobility direction (see Fig. 1b), causing a decrease of distance between peaks. As a result, very closely neighbored peaks start to merge (see $P_{II}$ and $P_{III}$ in Fig. 1b), leading to a decrease of peak number. This rule persists for higher temperatures (see Fig. 1c for $T=20K$). For sufficiently high temperature, the number of peaks decreases to just one or two. As a result, the commonly used two carrier model will also become suitable. As show in Ref.^28^, the mismatch between two carrier model fitting and experimental result indeed gradually disappears as temperature increases.

**Figure 3:**
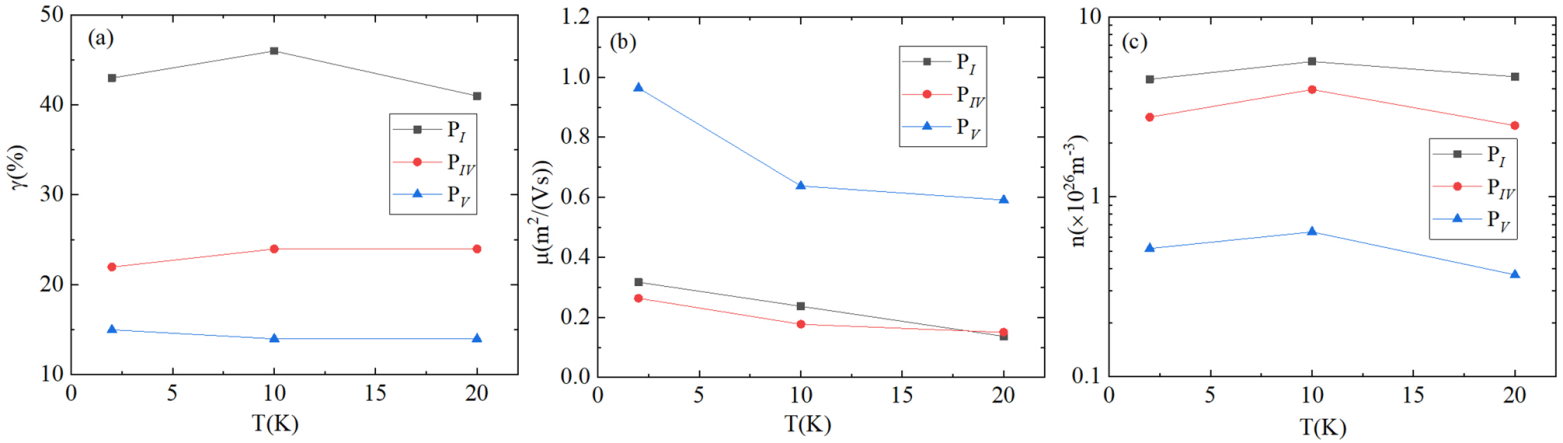
The ratio of (**a**) conductivity contribution $\gamma$, (**b**) mobility $\mu$, and (**c**) carrier concentration *n* as a function of temperature *T* for $P_I$ (black squares), $P_{IV}$ (red circles), $P_{V}$ (blue triangles), respectively.

An advantage of MSA is that it can analyse carrier’s temperature dependence properties. Note that, the accuracy of MS is sensitive to mobility as higher mobility carriers are more impressionable to external field. Therefore, here, we limit our analysis to high mobility carriers only, i.e., neglecting $P_{II}$ and $P_{III}$. In Fig. 3, we plot ratio of conductivity contribution $\gamma$, mobility $\mu$, and carrier concentration *n* as a function of temperature for $P_I$, $P_{IV}$, $P_{V}$, respectively. The ratio of conductivity contribution $\gamma$ and carrier concentration *n* are robust to temperature change (see Fig. 3a,c), whereas, the mobility of each peak decreases with increasing temperature (see Fig. 3b), especially for the Dirac-like pocket $P_{V}$. The information of all the peaks for different temperature are summarized in Table 1.

**Figure 4:**
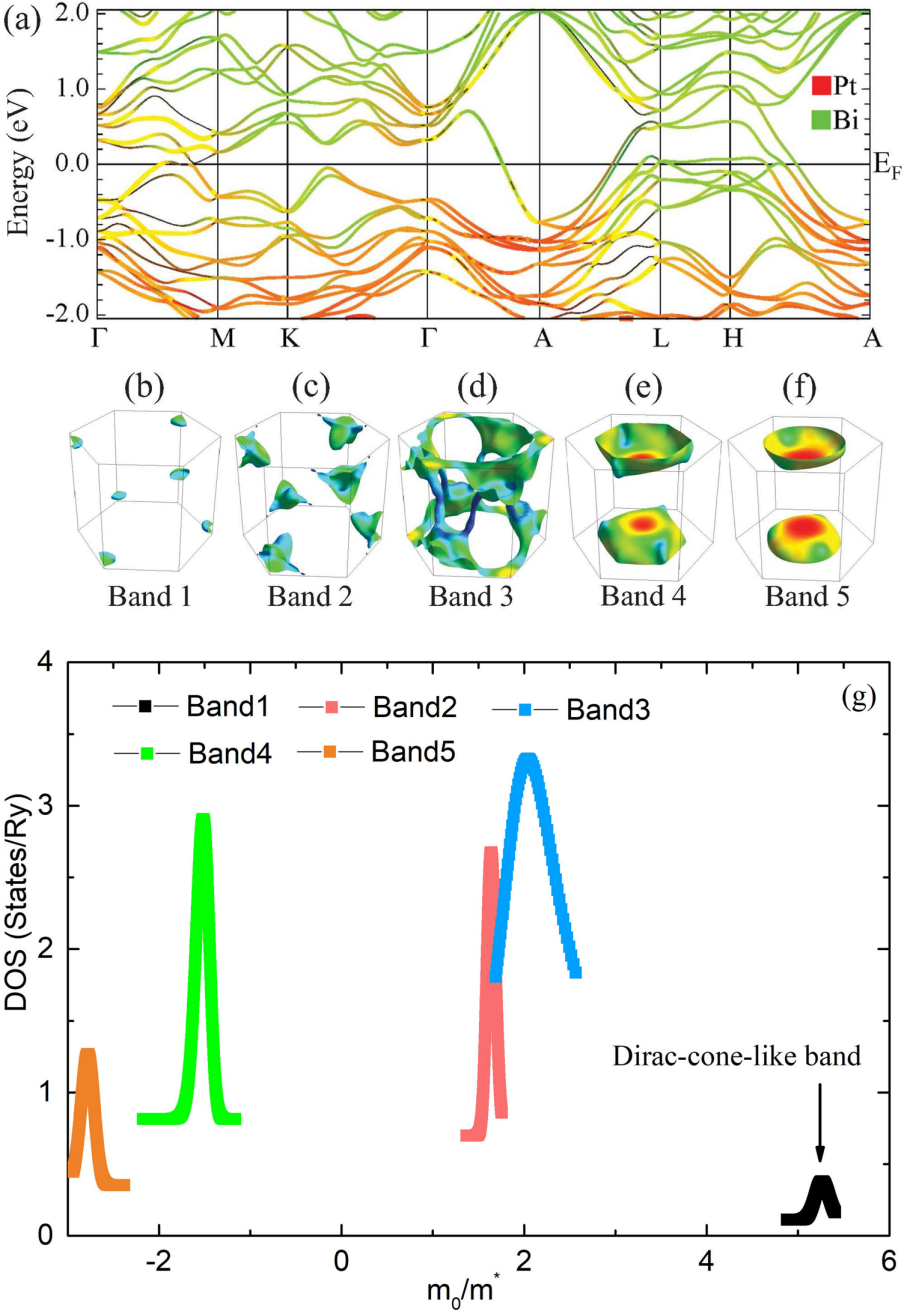
(**a**) The calculated electronic band structure of PtBi$_2$. The green and red lines indicates the Bi-*p* and Pt-*d* orbital contributions, respectively. (**b**–**f**) Three-dimensional Fermi surfaces of PtBi$_2$ for each band in the first Brillouin zone. (**g**) The DOS as a function of effective mass. Five bands including a Dirac-cone-like band (black line) is identified.

**Table 1:** Information of each peaks separated by MEMSA: contribution to the conductivity $\gamma$, average mobility $\mu$, and change concentration n.

T		electrons		holes		
		P$_I$	P$_{II}$	P$_{III}$	P$_{IV}$	P$_V$
2K	$\gamma$	43%	6%	9%	22%	15%
	$\mu$ (m$^2$/(Vs))	0.317	0.070	0.077	0.264	0.964
	*n* (m$^{-3}$)	$4.5\times 10^{26}$	$2.8 \times 10^{26}$	$3.9\times 10^{26}$	$2.8\times 10^{26}$	$5.2\times 10^{25}$
		P$_I$	P$_{II}$+ P$_{III}$		P$_{IV}$	P$_V$
10K	$\gamma$	46%	8%		24%	14%
	$\mu$ (m$^2$/(Vs))	0.237	0.003		0.177	0.637
	*n* (m$^{-3}$)	$5.7\times 10^{26}$	$7.8\times 10^{27}$		$3.9\times 10^{26}$	$6.4\times 10^{25}$
20K	$\gamma$	41%	18%		24%	14%
	$\mu$ (m$^2$/(Vs))	0.137	0.017		0.150	0.590
	*n* (m$^{-3}$)	$4.7\times 10^{26}$	$1.6\times 10^{27}$		$2.5\times 10^{26}$	$3.7\times 10^{25}$

## Discussion

The MS allows us to explain many physics phenomena. For example, the sign change phenomenon of Hall conductivity $\sigma _{xy}$: At low field $B\ll 1/\mu$, conductivity is the summation of all carriers’ contribution. After increasing field, high mobility carriers with $\mu B\gg 1$ are gradually localized, i.e., only low mobility carriers are responding for conducting. Therefore, the winner of the competing between electron and hole conductivity contribution may swap, which leads to change of Hall conductivity’s sign. For PtBi$_2$, as was discussed previously, the conductivity contribution for electrons and holes is comparable ($48\%$ for electrons and $52\%$ for holes) at zero field. Contribution of holes are slightly larger, so the Hall conductivity sign is positive. As field increases, the Dirac-like hole pocket $P_V$, which has the highest mobility, will first be localized. Therefore, holes’ contributions decreases faster than that of electron. Once the electrons win the competition, the sign of Hall conductivity changes.

We further compare our MEMSA results with electron band structure obtained by first principle calculation. The band structure of PtBi$_2$ is calculated using the full-potential linearized augmented plane wave (FP−LAPW) method implemented in the WIEN2K code. In Fig. 4a, we show the fat band of PtBi$_2$ with orbital characters. There are five bands crossing Fermi level $E_F$, hybridized from Pt *d* and Bi *p* orbitals. A distinct Dirac cone near $E_F$ locates at the *L* point. Further band analysis demonstrates that, there are three hole-like bands which construct hole pockets locate around the Brillouin zone corner (band 1, 2 and 3), and two electron-like bands which construct bowl-shaped electron pockets centered around *A* point (band 4 and band 5), as shown in Fig. 4b–f.

We further calculate the density of state (DOS) at $E_F$ as a function of $m_0/m^*$ ($m_0$ and $m^*$ are free-electron mass and quasiparticle effective mass, respectively). As shown in Fig. 4g, the DOS also has peak structures: including three hole-like and two electron-like peaks. Interesting, there is a small peak corresponding to band I, whose effective mass ($m^*\approx 0.2m_e$) is small, consisting with the Dirac-like band $P_V$ in Fig. 1.

## Conclusions

In summary, we use MEMSA to study carrier property of PtBi$_2$. We demonstrate that MEMSA not only can identify carrier type, and get band number, but also can calculate many other important information, including mobility, concentration, and conductivity contribution. Comparing with integration methods or multi-carrier fitting methods, MEMSA can spontaneously fit both experimentally measured conductivity and Hall conductivity, and produce high resolution results. For PtBi$_2$, three hole pockets and two electron pockets are identified, which agree with previous reports^30,33^, as well as our first principle calculation. The higher resolution of MS enable us to deeply analysis carrier properties: Carrier’s mobility decreases as temperature increases, whereas, carrier concentration is rather robust to temperature change. Our results explains the sign change phenomenon of Hall conductivity: High mobility carriers are localized at high field and have no contribution to conductivity. Moreover, MEMSA shows a small hole pocket with very high mobility, which agrees with the little hole-like pocket with small effective mass find by first principle calculation. These features show that this small pocket is very likely corresponding to previously reported Dirac Fermions^31^. Our results show that MEMSA is a useful tool to study band structure and carrier properties of large magnetoresistance materials.

## Supplementary information

Supplementary information.

## References

[1] Kamihara, Y., Watanabe, T., Hirano, M. & Hosono, H. Iron-based layered superconductor La[O$$_{{1-x}}$$F$$_{x}$$]FeAs (*x* = 0.05$$-$$0.12) with $$T_c$$ = 26 K. *J. Am. Chem. Soc.* **130**, 3296–3297, 10.1021/ja800073m (2008). 10.1021/ja800073m 18293989

[2] Singh DJ, Du M-H (2008). Density functional study of LaFeAsO$$_{{1-x}}$$F$$_{x}$$: a low carrier density superconductor near itinerant magnetism. Phys. Rev. Lett..

[3] Kasahara S (2010). Evolution from non-Fermi- to Fermi-liquid transport via isovalent doping in BaFe$$_{2}$$( As$$_{{1-x}}$$P$$_{x}$$)$$_{2}$$ superconductors. Phys. Rev. B.

[4] Cheng, P. *et al.* Hall effect and magnetoresistance in single crystals of NdFeAsO$$_{{1-x}}$$F$$_{x}$$ ( x = 0 and 0.18). *Phys. Rev. B* **78**, 134508, 10.1103/PhysRevB.78.134508 (2008).

[5] Ali MN (2014). Large, non-saturating magnetoresistance in WTe$$_{2}$$. Nature.

[6] Tafti FF, Gibson QD, Kushwaha SK, Haldolaarachchige N, Cava RJ (2016). Resistivity plateau and extreme magnetoresistance in LaSb. Nat. Phys..

[7] Fallah Tafti, F. *et al.* Temperature$$-$$field phase diagram of extreme magnetoresistance. *Proc. Natl. Acad. Sci.* **113**, E3475–E3481, 10.1073/pnas.1607319113 (2016). 10.1073/pnas.1607319113 PMC4922163 27274081

[8] Liang T (2015). Ultrahigh mobility and giant magnetoresistance in the Dirac semimetal Cd$$_{3}$$As$$_{2}$$. Nat. Mater..

[9] Luo X (2018). Origin of the extremely large magnetoresistance in topological semimetal PtSn$$_{4}$$. Phys. Rev. B.

[10] Weng H, Fang C, Fang Z, Dai X (2016). Topological semimetals with triply degenerate nodal points in $$\theta $$-phase tantalum nitride. Phys. Rev. B.

[11] Armitage NP, Mele EJ, Vishwanath A (2018). Weyl and Dirac semimetals in three dimensional solids. Rev. Mod. Phys..

[12] Potter AC, Kimchi I, Vishwanath A (2014). Quantum oscillations from surface Fermi arcs in Weyl and Dirac semimetals. Nat. Commun..

[13] Bradlyn, B. *et al.* Beyond Dirac and Weyl fermions: Unconventional quasiparticles in conventional crystals. *Science* **353**, aaf5037, 10.1126/science.aaf5037 (2016). 10.1126/science.aaf5037 27445310

[14] Zhu Z, Winkler GW, Wu Q, Li J, Soluyanov AA (2016). Triple point topological metals. Phys. Rev. X.

[15] Weng H, Fang C, Fang Z, Dai X (2016). Coexistence of Weyl fermion and massless triply degenerate nodal points. Phys. Rev. B.

[16] Watson MD (2015). Dichotomy between the hole and electron behavior in multiband superconductor FeSe probed by ultrahigh magnetic fields. Phys. Rev. Lett..

[17] Lv BQ (2017). Observation of three-component fermions in the topological semimetal molybdenum phosphide. Nature.

[18] Kurita, N. *et al.* Pressure-induced superconductivity in EuFe$$_{2}$$As$$_{2}$$ without a quantum critical point: Magnetotransport and upper critical field measurements under high pressure. *Phys. Rev. B* **88**, 10.1103/PhysRevB.88.224510 (2013).

[19] Ishida, S. *et al.* Manifestations of multiple-carrier charge transport in the magnetostructurally ordered phase of BaFe$$_{2}$$As$$_{2}$$. *Phys. Rev. B* **84**, 10.1103/PhysRevB.84.184514 (2011).

[20] Huynh KK, Tanabe Y, Tanigaki K (2011). Both electron and hole Dirac cone states in Ba ( FeAs )$$_{2}$$ confirmed by magnetoresistance. Phys. Rev. Lett..

[21] McClure JW (1956). Field dependence of magnetoconductivity. Phys. Rev..

[22] McClure JW (1958). Analysis of multicarrier galvanomagnetic data for graphite. Phys. Rev..

[23] Beck WA, Anderson JR (1987). Determination of electrical transport properties using a novel magnetic field-dependent Hall technique. J. Appl. Phys..

[24] Rothman J, Meilhan J, Perrais G, Belle J-P, Gravrand O (2006). Maximum entropy mobility spectrum analysis of HgCdTe heterostructures. J. Electron. Mater..

[25] Huynh KK (2014). Mobility spectrum analytical approach for intrinsic band picture of Ba(FeAs)$$_{2}$$. New J. Phys..

[26] Huynh KK (2014). Electric transport of a single-crystal iron chalcogenide FeSe superconductor: Evidence of symmetry-breakdown nematicity and additional ultrafast Dirac cone-like carriers. Phys. Rev. B.

[27] Pei QL (2018). Mobility spectrum analytical approach for the type-II Weyl semimetal $$T_d$$-MoTe$$_{2}$$. Appl. Phys. Lett..

[28] Xu CQ (2016). Synthesis, physical properties, and band structure of the layered bismuthide PtBi$$_{2}$$. Phys. Rev. B.

[29] Yang X (2016). Giant linear magneto-resistance in nonmagnetic PtBi$$_{2}$$. Appl. Phys. Lett..

[30] Gao W (2018). A possible candidate for triply degenerate point fermions in trigonal layered PtBi$$_{2}$$. Nat. Commun..

[31] Thirupathaiah S (2018). Possible origin of linear magnetoresistance: Observation of Dirac surface states in layered PtBi$$_{2}$$. Phys. Rev. B.

[32] Wu B, Barrena V, Suderow H, Guillamón I (2020). Huge linear magnetoresistance due to open orbits in $$\gamma $$-PtBi$$_{2}$$. Phys. Rev. Res..

[33] Jiang W (2020). Electronic structure of non-centrosymmetric PtBi$$_{2}$$ studied by angle-resolved photoemission spectroscopy. J. Appl. Phys..

[34] Kiatgamolchai, S. *et al.* Mobility spectrum computational analysis using a maximum entropy approach. *Phys. Rev. E* **66**, 10.1103/PhysRevE.66.036705 (2002). 10.1103/PhysRevE.66.036705 12366296

[35] Gao, W. *et al.* Extremely large magnetoresistance in a topological semimetal candidate pyrite PtBi$$_{2}$$. *Phys. Rev. Lett.* **118**, 10.1103/PhysRevLett.118.256601 (2017). 10.1103/PhysRevLett.118.256601 28696743

