# Supplementary material for: Transport property of multi-band topological material PtBi2 studied by maximum entropy mobility spectrum analysis (MEMSA)
Source: Sci Rep. 2021 Mar 18;11:6249. doi: 10.1038/s41598-021-85364-6 (PMC7973776; doi:10.1038/s41598-021-85364-6)
Supplement: Supplementary file 1 — Supplementary information. [file 41598_2021_85364_MOESM1_ESM.pdf]

Supplementary Materials for Transport Property of Multi-band Topological Material $PtBi_2$ Studied by Maximum Entropy Mobility Spectrum Analysis (MEMSA)

Haijun Zhao[1,*], Wenchong Li[1], Yue Chen[1], Chunqiang Xu[1], Bin Li[2,+], Weidong Luo[3,4], Dong Qian[3,4], and Zhixiang Shi[1,†]

[1]School of Physics, Southeast University, Nanjing 211189, China.
[2]Information Physics Research Center, Nanjing University of Posts and Telecommunications, Nanjing 210023, China
[3]Key Laboratory of Artificial Structures and Quantum Control (Ministry of Education), School of Physics and Astronomy, Shanghai Jiao Tong University, Shanghai 200240, China
[4]Tsung-Dao Lee Institute, Shanghai Jiao Tong University, Shanghai 200240, China
[*]Correspondence to haijunzhao@seu.edu.cn
[+]Correspondence to libin@njupt.edu.cn
[†]Correspondence to zxshi@seu.edu.cn

ABSTRACT

Electrical transport of both longitudinal and transverse directions carries rich information. Mobility spectrum analysis (MSA) of conducting tensor is capable of extracting carriers' type, concentration and mobility. Using a numerical method based on maximum entropy principle, i.e., Maximum Entropy Mobility Spectrum Analysis (MEMSA), a mobility spectrum composed of three hole-pockets and two electron-pockets is obtained for β-type $PtBi_2$. Specially, we found a small hole pocket with very high mobility, which is very likely corresponding to Dirac Fermions. Benefiting from the sufficiently high resolution, the temperature dependence of each pocket is analyzed. We further compared the results with band structure obtained by our first principle calculation. The present results prove MEMSA is a useful tool of extracting carries' information.

Comparing numerical method base on max entropy principle and KK principle transition method

In Fig. S1(a), we show the Mobility spectrum of $BaFe_2As_2$ shown in Ref.[1], which was obtained by KK principle transition. Our numerical MS result calculated from the same experimental date is shown in Fig. S1(b). The numerical fit of our MEMSA result to the conductor tensor is shown in the inset of Fig. S1(b). It is clear that our numerical result have similar peak structures comparing with previous reported work, but its resolution is much higher. Moreover, our result is capable of well fitting the experimental data.

Fitting to the experimental data

In Fig. S2, we show our experimental data of $PtBi_2$, as well as its numerical fit by MEMSA data. Benefited from the max entropy principle, the numerical fit acts like a smoothed result of the experimental data, therefore one do not need to smooth the experimental data before numerical calculation. Comparing with two carrier model shown in Ref.[2], which only fits well for high temperature case, MS is also capable of fitting low temperature data.

References

1. Huynh, K. K. *et al.* Mobility spectrum analytical approach for intrinsic band picture of $Ba(FeAs)_2$. *New J. Phys.* **16**, 093062, DOI: 10.1088/1367-2630/16/9/093062 (2014).

2. Xu, C. Q. *et al.* Synthesis, physical properties, and band structure of the layered bismuthide $PtBi_2$. *Phys. Rev. B* **94**, 165119, DOI: 10.1103/PhysRevB.94.165119 (2016).

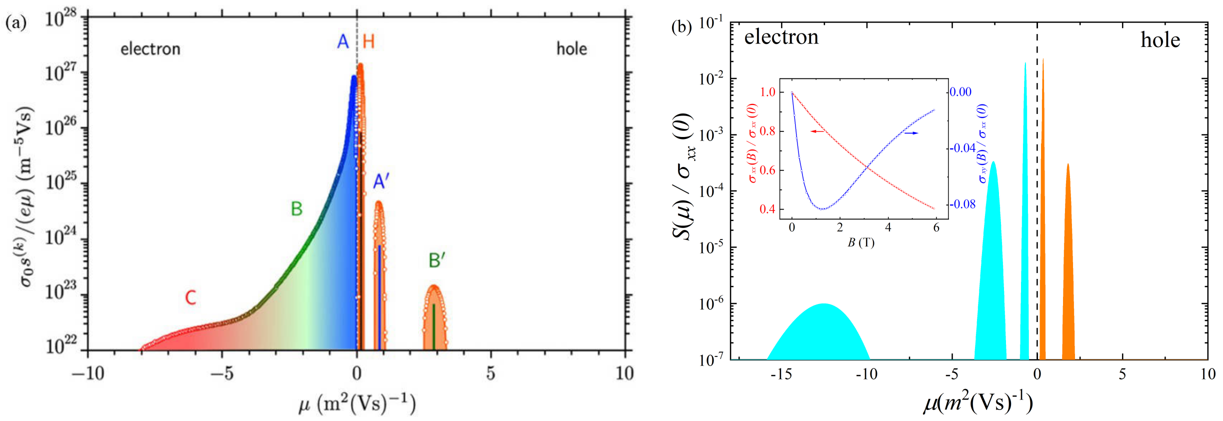

Figure S1. (Coloronline)(a) Mobility spectrum of $BaFe_2As_2$ taken from Ref.[1], which was obtained by KK principle transition.(b) Main panel: Mobility spectrum obtained by our numerical method. (b) Inset: Experimental curve of conductor tenser (dots) and its numerical fit (lines).

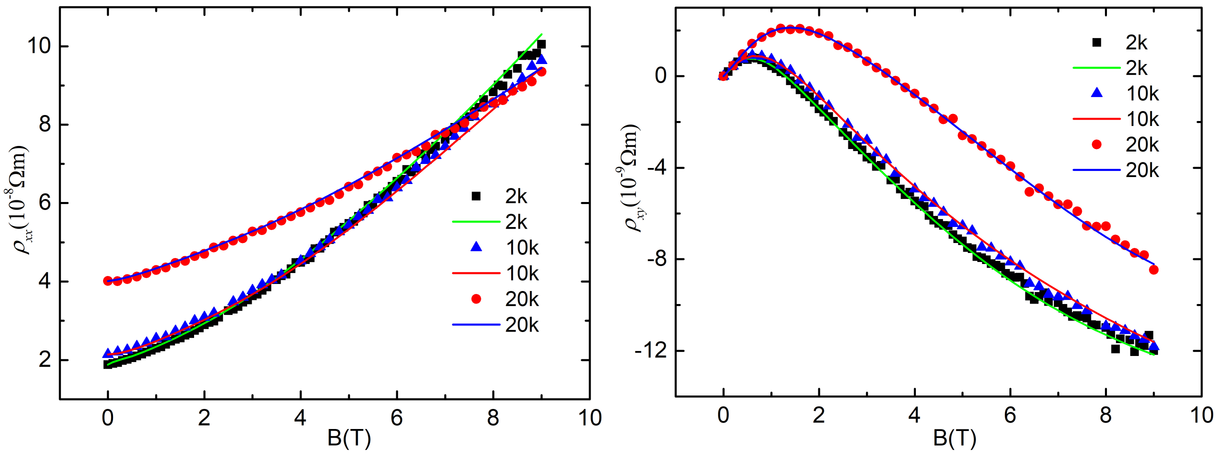

Figure S2. (Coloronline)The experimentally measured resistance (left panel) and Hall resistance (right panel) for temperature $T = 2K$ (black squares), $T = 10K$ (blue triangles), and $T = 20K$ (red circles), as well as their numerical fitting(solid lines) by MEMSA.
